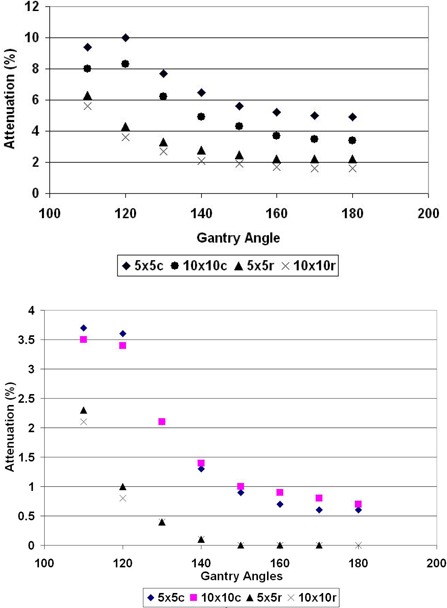# Erratum: “Determination of the photon beam attenuation by the BrainLAB imaging couch: angular and field size dependence”

**DOI:** 10.1120/jacmp.v12i1.3474

**Published:** 2010-11-23

**Authors:** Njeh et al.

**Affiliations:** ^1^ Radiation Oncology Department Texas Oncology Tyler TX 75702 USA

In the original article, Fig. 3: Gantry dependence of the transmission of 6 MV photon beam to the BrainLAB's couch and Fig. 4: Gantry dependence of the transmission of 18 MV photon beam to the BrainLAB's couch, were identical. We have presented the two corrected figures.